# TGF-β/Smad Signalling in Neurogenesis: Implications for Neuropsychiatric Diseases

**DOI:** 10.3390/cells10061382

**Published:** 2021-06-03

**Authors:** Lih-Fhung Hiew, Chi-Him Poon, Heng-Ze You, Lee-Wei Lim

**Affiliations:** Neuromodulation Laboratory, School of Biomedical Sciences, Li Ka Shing Faculty of Medicine, The University of Hong Kong, Hong Kong, China; u3005074@connect.hku.hk (L.-F.H.); chpoonac@connect.hku.hk (C.-H.P.); 17097461d@connect.polyu.hk (H.-Z.Y.)

**Keywords:** neurogenesis, TGF-β, smad, depression, stress

## Abstract

TGF-β/Smad signalling has been the subject of extensive research due to its role in the cell cycle and carcinogenesis. Modifications to the TGF-β/Smad signalling pathway have been found to produce disparate effects on neurogenesis. We review the current research on canonical and non-canonical TGF-β/Smad signalling pathways and their functions in neurogenesis. We also examine the observed role of neurogenesis in neuropsychiatric disorders and the relationship between TGF-β/Smad signalling and neurogenesis in response to stressors. Overlapping mechanisms of cell proliferation, neurogenesis, and the development of mood disorders in response to stressors suggest that TGF-β/Smad signalling is an important regulator of stress response and is implicated in the behavioural outcomes of mood disorders.

## 1. Introduction

The transforming growth factor-β (TGF-β) pathway consists of many genes involved in cell growth, differentiation, migration, and apoptosis [1,2]. TGF-β ligands bind to the TGF-β receptor kinase to form a complex that phosphorylates receptor-activated Smads (R-Smads), allowing recruitment of Smad4 that translocates to the nucleus to regulate transcriptional activity [3]. Smad-mediated TGF-β signalling is referred to as the canonical pathway. Notably, Smad3 is the primary molecule involved in canonical TGF-β signalling [4]. TGF-β also activates a myriad of other pathways, including the Erk, JNK, and MAPK pathways [5], which together are known as the non-canonical pathway. TGF-β is involved in a vast number of interactions and can have many roles depending on the cellular context. TGF-β has been found in neural progenitor cells, differentiating neurones, and mature neural cells. TGF-β exhibits both anti-tumour properties and tumourigenic properties [6] depending on the manner of its activation. Given that TGF-β is known to have proliferative effects in somatic cells [7], it was proposed that they would have a similar role in neural cells. Indeed, TGF-β induces cell cycle exit in murine hippocampal neurones [8] and has been associated with the loss of adult neurogenesis through arresting the proliferation of progenitor cells [9]. TGF-β also plays a function in various neurogenic processes, including the formation and elongation of axons [10], neurite growth [11], and initiation of neuronal migration [12]. Given the diverse roles of TGF-β and its function in the nervous system, it is clear that TGF-β signalling is also involved in neuroplasticity and neuroprotection.

Neuroplasticity broadly refers to the ability of the nervous system to responds to internal and external stimuli by reorganising its structure and function at the molecular, cellular, and organisational levels. These adaptations can be beneficial or harmful to the organism; consequently, neuropsychiatric disorders have been examined as a manifestation of deleterious neuroplasticity. Indeed, neuropsychiatric disorders have been characterised according to alterations in the limbic, fronto-striatal, and prefrontal circuits, which in turn generate disturbances in behaviour, cognition, and motivation [13]. These behavioural changes can manifest over long periods, meaning the course of treatments can be similarly drawn out. The improvements due to such treatments can also be lost over time, and each episode of illness increases the probability of relapse as the altered neural networks become more dysfunctional [14]. Neuroinflammation, neuronal survival, and proliferation are some of the factors that can negatively influence neuroplasticity. Furthermore, these processes can also be negatively affected by inflammation, as demonstrated by treatment with lipopolysaccharides from *E. coli* used to induce an immune response [15]. Neuroinflammation has also been implicated in several nervous system diseases, including multiple sclerosis, Alzheimer’s Disease, Parkinson’s Disease, and major depression [16]. Although inflammation is commonly associated with physical injury or infection, stress can also trigger the release of inflammatory cytokines [17], such as Interleukin-1β, Interleukin-6, and tumour necrosis factor-α (TNF-α), that interfere with the production of neural growth factors. Meanwhile, preclinical studies inhibiting interleukin-1 in mouse model showed effective reversal of this effect and alleviated stress-induced behavioural changes [18,19].

Evidence from previous work suggests an association between TGF-β signalling and the phenomena of neuroplasticity and neurogenesis, as well as their combined effects on mood disorders [20,21,22]. Based on these findings, TGF-β signalling mediated by Smad3 has been hypothesised to play an important role in neurogenesis in the hippocampus, and has been implicated in the development of mood disorders and the manifestation of depressive and anxiety disorders. Previous studies on mood disorders were largely based on neuroplasticity mechanisms, however, the underlying pathways are not well understood and relatively underexplored [23,24]. Thus, we hypothesise the neurogenic mechanisms of depression and anxiety involve TGF-β and its signalling molecules.

## 2. Non-Canonical Signalling

Non-canonical TGF-β signalling refers to molecular events that occur independent of the SMAD pathway. TGF-β activated kinase 1 (TAK1) is an important effector in non-canonical TGF-β signalling. TAK1 initiates a signalling cascade that activates c-Jun N-terminal Kinases (JNK) and p38/mitogen activated protein kinase (MAPK) in response to TNF/TGF-β signalling, as well as activates the IkB Kinase (IKK) and Nuclear Factor-κB (NF-κB) pathways. TAK1 is an important regulator of innate and adaptive immune responses [25], and is essential for the survival of haematopoietic cells and hepatocytes [26]. TAK1 is highly expressed in the brain [27] and is assumed to play a role in neural functions. Indeed, TAK1 was shown to be important in axon growth, as neurones with knockdown of TAK1 had significantly shorter axons than normal neurones. TAK1 also rescued axonal growth in the presence of JNK inhibitor SP600125. Furthermore, TAK1-negative mice exhibited embryonic lethality due to defects in the brain, supporting the essential role of TAK1 in brain development [28]. TAK1 is also a major signalling partner of TGF-β, as TGF-β receptors could only induce low levels of JNK phosphorylation in TAK1-negative cells [28].

The MAPK signalling pathway has been shown to be involved in neuronal differentiation and axon growth [29]. The MAPK pathway has also been implicated in the development of depression [30], as the inhibition of MAPK signalling inducing depressive-like behaviours [31]. Indeed, some pharmacological treatments for depression activate the MAPK signalling pathway [32]. It was shown that MAPK differentially affected mice depending on age, with juvenile mice spending significantly more time in the open arms of the elevated plus maze (EPM), indicating ablated anxiety compared with wildtype mice [33]. The involvement of MAPK signalling in mood disorders was further studied by knockout of Braf, an upstream effector of MAPK. Adult mice lacking Braf showed greater passivity in the forced swim test, indicating greater depressive behaviours [33]. Taken together, these findings indicate MAPK pathway has differential effects across the lifespan, highlighting the potential of targeting MAPK signalling in regulating the pathogenesis of mood disorders.

The c-AMP response element binding protein (CREB) is involved in the TGF-β pathway, as TGF-β was shown to activate extracellular elements that activate CREB [34]. TGF-β was also able to directly induce phosphorylation of CREB, as shown by increased phosphorylation of CREB in hippocampal cells treated with TGF-β2 compared with the control, implying that CREB can mediate the long-term effects of TGF-β2 [35]. CREB has also been implicated in hippocampal functions such as memory [36] and has been targeted by antidepressants [37]. Loss of CREB function in the brains of mice was shown to increase neurogenesis and abolish depressive-like behaviour in assays, such as the forced swim test [38]. Various other studies showed CREB is important in cell survival and maturation in the hippocampus [39,40], and can improve the antidepressant response [41]. Further studies are needed to understand the role of CREB in neurogenesis.

## 3. Canonical Signalling in Neurogenesis

TGF-β has been shown to play a role in inflammation and promote cell survival, induce apoptosis, and initiate proliferation and differentiation [2,42]. In mammals, TGF-β exists as three molecular isoforms (TGF-β1, β2, and β3) found in the cerebral cortex [43], olfactory epithelium [44], adult astrocytes, neurones, and microglia [45]. TGF-β is involved in the initiation of cell cycle exit and neuronal differentiation, among other functions. TGF-β is also required for neuronal survival. This is consistently supported by in vitro and in vivo data, which demonstrated lower survivability of TGF-β knockout neurones [46], and embryonic lethality, increased neuronal apoptosis and other abnormalities such as reduced synaptic integrity and microgliosis, observed in TGF-β-null mice [46,47]. Taken together, these results indicate that TGF-β plays a vital role in neuronal survival and microglial activation. Given that Smad3 is an important effector of TGF-β function, the implication of these findings on Smad3 warrants further research to delineate its functions in the brain.

Despite extensive research on the effect of TGF-β on neurogenesis (Table 1), the relationship between Smad3 signalling and neurogenesis is not well understood. Previous research on Smad3 deficiency indicated that Smad3 signalling was important in neurogenesis, given that Smad3 was found to be abundantly expressed in neurogenic zones [48,49,50]. The role of Smad3 in neurogenesis is further corroborated by the finding that showed colocalization of Smad3 transcript with mature neuron marker neuronal nuclei (NeuN) in the dentate gyrus of hippocampus [51]. Moreover, Smad3-null mice showed disrupted neuronal proliferation and migration [49] and expressed significantly less neurones in the dentate gyrus compared with wildtype mice [49]. Conversely, Smad3-null mice exhibited a series of alterations, for instance impaired hippocampal neurogenesis, reduced newborn neurone survival and elimination of long-term potentiation (LTP) in the medial perforant pathway by facilitating gamma-amino butyric acid (GABAergic) signalling [51,52]. These results provided evidence supporting the broad potential of Smad3 in modulating neurogenesis. Interestingly, these effects appeared to be region-specific, as increased progenitor cell population in the rostral hippocampus and intact LTP in the Schaffer collateral pathway were also found in Smad3-null mice [51]. Similarly, increased levels of TGF-β reduced neurogenesis in vitro and in vivo at rates largely similar to that in Smad3-deficient animals [9,30,53,54]. Contrary to these findings, it was shown that knockout of TGF-β receptor activin receptor-like kinase 5 (ALK5) resulted in decreased neurogenesis, whereas upregulation of ALK5 resulted in greater neurogenesis and improved memory functions [55]. This indicates that the components in the canonical pathway, especially Smad3, potentially plays a role in memory and cognitive functions and may regulate neurogenesis differentially in various parts of the brain. Furthermore, Smad3 signalling in neurones and astroglia were found to differentially regulate dendritic spine growth [56], whereas its inhibition leads to increased susceptibility to neuronal apoptosis [57], potentially increasing the risk of Parkinson’s disease [25] and neurodegeneration [26]. However, the specific effects of Smad3 on behaviour have yet to be studied.

**Figure 1:**
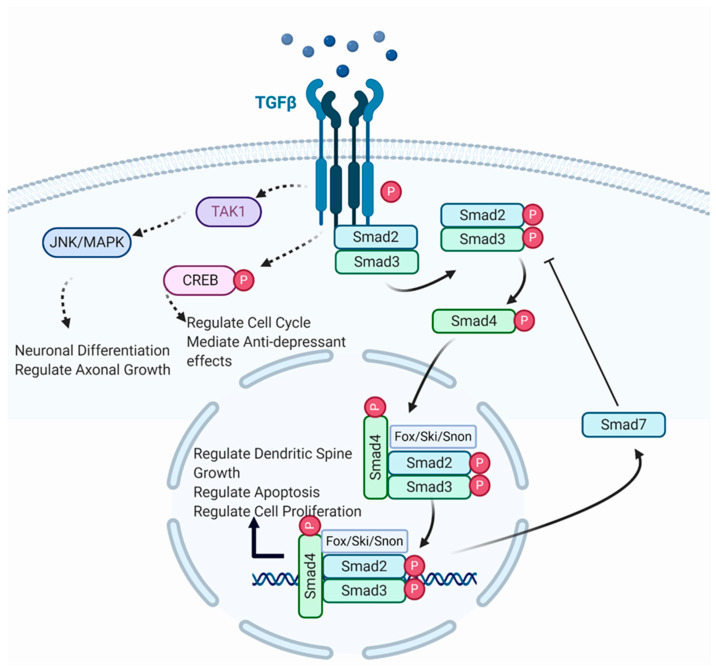
Illustration of canonical and non-canonical TGF-β signalling. TGF-β ligand binds with TGF-β receptor to form a complex (Created with BioRender.com, accessed on 19 April 2021). The receptor-induced phosphorylation of R-Smads leads to binding with cytoplasmic Smad2/3. Phosphorylated Smads form a complex with Smad4, which translocates to the nucleus where it binds with various transcription factors for gene transcription. Smad complexes initiate a negative feedback loop, leading to Smad7 inhibition of further phosphorylation of R-Smads. TGF-β receptors also phosphorylate TAK1 and CREB, which regulate neuronal differentiation, axonal growth, cell cycle progression, and anti-depressant effects, respectively.

Besides interacting with TGF-β, Smad3 also interacts with a host of other signalling molecules such as its close analogue Smad2 (Figure 1). Although both proteins share 91% amino acid sequence, they recruit different cofactors and thus target different transcription pathways [43]. Nevertheless, they have been shown to share redundant roles in certain contexts, particularly [44,45,48,66]. Both Smad2 and Smad3 were found to cooperate and antagonize targets simultaneously, for example, Smad3 activates its targets while antagonizing Smad2 targets. This is because Smad2 targets only respond to Smad2 homodimers, whereas Smad3 targets respond to both Smad3 homodimers and heterodimers, thus increased co-expression of Smad2 and Smad3 leads to greater activation of Smad3 targets but inactivation of Smad2 targets [48]. In terms of neurogenic mechanisms, knockdown of Smad3 reduced neurogenesis, whereas knockdown of Smad2 increased neurogenesis [48]. Silencing of Smad3 in aged mice by shRNA resulted in greater neurogenesis, indicating that Smad3 plays a significant role in hippocampal degeneration in old age [49]. This suggests that Smad2 would inhibit the pro-neurogenic effects of Smad3, whereas knockout of Smad3 would result in elevated Smad2 processes, resulting in an overall reduction of neurogenesis.

TGF-β binds to the type II receptor that in turn forms a complex that phosphorylates Smad3. TGF-β receptors were found to be expressed on new cells in the neurogenic region of the dentate gyrus and the subventricular zone [49]. Similarly, Smad3 was also found to be distributed in neurogenic zones, whereas Smad3-null mice exhibited markedly decreased Bromo-deoxyuridine (BrdU)-positive cells in the dentate gyrus and subventricular zone. Both Smad3 and Smad2 pathways are activated by activin in a manner similar to activation by TGF-β. It was found that activin A was upregulated in the dentate gyrus and the CA1 region of the hippocampus following chronic paroxetine treatment [56]. Additionally, direct injection of activin A into mouse dentate gyrus significantly reduced immobility in the forced swim test, indicating it can exert antidepressant-like effects [56]. Moreover, there was increased neuronal survival and development in rat hippocampal cell cultures treated with activin. Activin also affected dendritic spine growth by modulating actin dynamics [57]. Taken together, these results imply a possible relationship between TGF-β and activin signalling. The above findings also suggest that Smads could be worth investigating, as it remains unclear how activin interacts with downstream signalling molecules to exert its antidepressant effects.

## 4. TGF-β Signalling in Epigenetics

Smad signalling has been implicated in epigenetic functions such as Jumonji domain-containing protein D3 (JMJD3). Knockdown studies showed that spinal cord [67] and retinal [68] development are regulated by JMJD3. JMJD3 is also expressed in neural stem cells as well as doublecortin expressing neuroblasts. Postnatal JMJD3-null mice experienced significantly less neural growth and exhibited neuroblast migration disturbances [62], indicating JMJDs are required in neurogenesis. Adult neurogenesis was found to require JMJD3, as neural stem cells (NSCs) lacking JMJD3 had stunted neurogenesis and impaired differentiation with reduced oligodendrocyte production [62]. Moreover, JMJD3 knockdown impaired TGF-β signalling in NSCs. Further examination of the relationship between TGF-β and JMJD3 revealed that the functions of TGF-β in development, differentiation, and apoptosis were dependent on JMJD3 [63]. Additionally, Smad3 function was also found to involve JMJD3. Co-immunoprecipitation assays revealed that phosphorylated Smad3, but not Smad2, interacted with JMJD3. Genome-wide analysis also showed that Smad3 and JMJD3 expression overlapped at transcriptional start sites, indicating that JMJD3 is involved in Smad3 activation of gene transcription.

Growth arrest and DNA-damage inducible protein 45 (Gadd45) is another epigenetic regulator that is affected by the TGF-β signalling pathway, as shown by blocking of TGF-β and TrkB leading to the overexpression of Gadd45 in vitro [69]. Gadd45 functions as a modulator of hippocampal stem cell proliferation, and its deletion was shown to prevent demethylation of brain-derived neurotrophic factor (BDNF) leading to disrupted neurogenesis [61]. Inactivation of Smad4, which is a core component of Smad signalling, increased Gadd45 expression, implicating TGF-β/Smad interactions in the neurogenic function of Gadd45. The expression of Gadd45 was shown to increase when TGF-β receptor function was impaired, and this effect was amplified by simultaneously blocking TrkB signalling. These data indicate that TGF-β signalling is involved in DNA demethylase expression by suppressing Gadd45 expression. Further DNA methylation and gene expression analyses in the mouse genome following potassium chloride treatment revealed increased expression of certain genes, with at least six genes associated with psychiatric diseases such as autism and depression. Induction of depression in an animal model resulted in decreased Gadd45 expression in the hippocampus and prefrontal cortex, accompanied by a decrease in TGF-β2 and TGF-β3 expressions [69]. These findings show that TGF-β signalling plays a role in the regulation of epigenetic mechanisms. Given that Smad4, which operates in tandem with Smads2/3, is able to affect Gadd45 expression, the effects of impaired Smad3 phosphorylation may shed more light on the relationship between TGF-β and Gadd45.

## 5. Neurogenesis in Neuropsychiatric Disorders

Neurogenesis is hypothesised to play a role in depression via changes to the rate of neurogenesis, specifically the deleterious effects from stress-related neurogenesis in the dentate gyrus. Neurogenesis in the dentate gyrus can impact behavioural output and the efficacy of antidepressant treatments. New neurones heavily affects hippocampal circuitry and behaviour. Clinical evidence shows hippocampal shrinkage is linked to depression. In the pre-clinical stage, depressive behaviour results in a host of adverse effects on hippocampal neurogenesis, including reduced proliferation of neural stem cells and reduced neuronal survival. Furthermore, antidepressant treatment in rats induced hippocampal neurogenesis over time, in a pattern that mirrors the delay in antidepressant efficacy in human subjects [70]. One could associate neurogenesis with cellular reparation and plasticity, as these events have been shown in animal models given antidepressant treatments, electroconvulsive therapy, and stress reducing exercise. However, the capacity to draw a causal relationship between hippocampal neurogenesis and depression remains elusive as other studies showed a loss of neurogenesis does not necessarily lead to the development of depression [71] and stress does not always decrease neurogenesis [72].

Although a direct causal link between hippocampal neurogenesis and mood disorders seems unlikely, neurogenesis is nonetheless an important factor in the development of mood disorders. The neurogenic interactome model attempts to reconcile these opposing findings by taking into account various factors affecting neurogenesis. The neurogenic interactome considers the interactions between brain structures, the various functions of the hippocampus, and the heterogeneity of elements in the neurogenic niche [73]. For example, hippocampal connections to regions involved in stress responses and emotional memory indicates the involvement of adult neurogenesis in depression. The complex series of connections proposed by the interactome model can possibly explain the discrepancies between behaviour tests, in that each test induces different levels of stress and thus engages different components of the neurogenic interactome. The interactome also suggests that neurogenic mechanisms can respond to stressful stimuli in different ways, such as coping or adapting to stress, thus predictable stress might increase neurogenesis [74,75]. The concept of a neurogenic interactome provides a holistic framework to study the various factors in neurogenesis. It would be illuminating to determine the involvement of Smad3 in the interactome as well as the behavioural effects of disturbances in Smad3 signalling.

The pathogenesis of mood disorders has been hypothesised to be related to neuroplastic changes, particularly neuroimmune processes such as neuroinflammation, which might affect the central and periphery nervous systems to impact the neurobiology of depression. Neuroinflammatory processes are suspected to exert deleterious effects on various pathways, such as the hypothalamic-pituitary-adrenal (HPA) axis, neurogenesis, neuroimmune response, and neurocircuitry by upregulating apoptotic functions. Various mechanisms have been put forward on how the immune system regulates neurogenesis and neuroplasticity, particularly the role of T-reg cells on enhancing hippocampal neurogenesis via upregulating glial cell-derived neurotrophic factors and TGF-β. It was shown that T-reg cells are attenuated during depression, whereas T-reg cells are increased with antidepressant treatments. However, behavioural studies in animals with suppressed neurogenesis but not exposed to stressors showed that neurogenesis by itself does not produce a depressive phenotype, as there were no differences in the behaviour between animals with ablated neurogenesis and controls [76].

## 6. Effects of Stress on TGF-β Signalling and Neurogenesis

Stress has been shown to induce anxiety and depressive disorders by disturbing the HPA axis-regulated release of glucocorticoids [77,78]. In particular, hippocampal inhibitory regulation of the HPA axis can be disturbed by chronic stress [79,80]. Observations in various species indicated that the decreased cell proliferation and neurogenesis in the dentate gyrus following exposure to stressors was related to elevated stress hormones [81,82,83]. Thus, it follows that chronic stress could exacerbate depression by altering neuroplasticity and neurogenesis. Furthermore, it was shown that anti-depressant treatments could reverse the reduced neurogenesis and cell proliferation. These studies also demonstrated a causal link between hippocampal plasticity and depressive disorders. However, the finding that decreased hippocampal neurogenesis failed to increase sensitivity to stress casts doubt on the causal relationship between neurogenic effects in the hippocampus and depression. Even so, the effects of stress still constitute a major factor in the development of depressive disorders. The expression of TGF-β was shown to be reduced in the hippocampus of stressed rats, which coincided with increased expression of inflammatory cytokines such as IL-1β and lowered expression of anti-inflammatory cytokines such as IL-10 [84]. Neurogenesis was also found to be impaired, as demonstrated by the level of BrdU-stained cells in the dentate gyrus of these rats, which indicates a potential relationship between decreased TGF-β signalling, loss of neurogenic function, and development of depressive-like symptoms. In vitro studies showed neurones exposed to increased cortisol levels had decreased TGF-β expression and reduced neurogenesis, supporting this relationship [85].

A connection between stress and neurogenesis has also been observed, in that a reduction of neurogenesis precipitated the development of stress-related disorders such as post-traumatic stress disorder [86]. This connection is reflected in several animal models such as social defeat paradigm and unpredictable chronic mild stress to mimic symptoms of depression and stress disorders [76]. Neurogenic functions in the dentate gyrus are known to be sensitive to stress, showing reduced cell proliferation in response to stress or to elevated serum glucocorticoid levels, but induced cell division with reduced glucocorticoid levels [87]. A loss of neurogenic function in the dentate gyrus inhibits regular functioning of the neural circuitry, and thus prevents the formation of new cognition and associations, perpetuating the development of depression [88]. Indeed, adult neurogenesis is capable of neuroplasticity, which facilitates the acquisition and separation of closely spaced memories [89]. It has been shown that decreased neurogenesis results in impaired pattern separation and learning, whereas increased neurogenesis improves them. Apart from the obvious functions in learning and memory, pattern separation may also be important in recognizing danger and stress [90]. Mice exposed to social stressors undergo increased neurogenesis, possibly indicating superior recognition of negative stimuli, whereas preventing neurogenesis diminished social avoidance [72]. Stress also affects the amygdala by disrupting endocannabinoid signalling, resulting in pathological anxiety [91]. Stress has also been shown to increase dendritic spine length in the basolateral amygdala, which may be indicative of spine instability during plasticity responses, and such morphological changes correspond with increased anxiety [92].

The hippocampus has been implicated in the development of depressive disorders. Neuroimaging studies and post-mortem examinations showed an association between reduced hippocampal volume and the duration of depression [93,94,95]. Hippocampal neurogenesis was also found to be necessary for the function of certain antidepressants. The effects of imipramine and fluoxetine were abolished when hippocampal neurogenesis was ablated by irradiation. The importance of hippocampal neurogenesis in drug treatments was further reinforced by the observation that antidepressants also increased hippocampal neurogenesis. Hippocampal activity can also affect neurotrophin release, as demonstrated by the release of TGF-β and BDNF following induced depolarization [69]. In animals exposed to prenatal stress and in patients suffering from childhood trauma, the hippocampus was shown to express greater amounts of FoxO, a proinflammatory transcription factor that responds to insulin signalling as well as oxidative stress [96]. The upregulation of FoxO1 in the presence of cortisol mediated cortisol-induced reduction of neuronal proliferation [97].

The amygdala has also been implicated in the pathogenesis of anxiety disorders, likely caused by hyperexcitability of neurones in the amygdala [98,99]. Further study on the amygdala revealed the endocannabinoid system is a key pathway that influences the regulation of stress and anxiety. Disruption of endocannabinoid signalling induced neurobehavioural effects similar to those produced by exposure to stress, such as increased activation of the HPA axis and heightened anxiety behaviours [100,101]. Interestingly, these effects could be replicated by direct disruption of endocannabinoid signalling in the amygdala [102]. Chronic stress via impaired LMO4 function resulted in halting endocannabinoid signalling in the amygdala, which caused anxiety behaviours, supporting the involvement of the amygdala in regulating anxiety states [92].

## 7. Discussion and Perspectives

The TGF-β pathway has been implicated in a wide range of processes. This is evident by reports showing increased TGF-β in circulation predisposes an individual to hypertension [103] and polymorphisms in TGF-β result in variable risk for the development of oesophageal cancer [104]. Intriguingly, TGF-β demonstrated paradoxical effects in oncology studies, in which TGF-β exhibited essential roles in tumorigenesis as well as tumour suppression. Histology on clinical samples of gastric carcinoma revealed that TGF-β itself gradually increases in expression as carcinoma progresses and is correlated with advanced stages of carcinoma while Smad molecules are associated with earlier stages [105]. Further substantiating the role of TGF-β in tumorigenesis is the finding that exogenous upregulation of TGF-β induces tumours with metastatic properties in zebrafish [106]. Interestingly, loss of TGF-β function in carcinomas appears to contribute to tumour suppressant properties [107,108]. Specifically, TGF-β exercises anti-proliferative effects through Smad signalling by increasing expression of Cyclin-dependent kinase (Cdk), which in turn results in cell cycle arrest [109]. Indeed, aberration in Smad function of gastric cancer cells renders TGF-β mediated growth inhibition ineffective [110]. The role that TGF-β plays in the cell cycle is no less apparent in the brain, directing axon growth during the developmental stage with cells lacking TGF-β failing to form axons [12]. Furthermore, the endogenous levels of TGF-β in circulation varies among the population, and this produces differences in phenotype. For instance, overexpression of TGF-β seems to heighten its antiproliferative effects, as the development of several cancers are ablated by increase of TGF-β [111,112,113]. These findings indicate that cell proliferation mechanisms can be affected through measured manipulation of TGF-β and its associated signalling molecules, as such there is potential in the TGF-β pathway modulation to alleviate disorders involving premature apoptosis and impaired cell proliferation.

TGF-β plays an important role in the central nervous system as a response to injury and trauma and functions as a signal to initiate cellular repair and protection [114,115]. TGF-β has been shown to be upregulated significantly following spinal lesioning and was observed in astrocytes, motor neurons, and surrounding epithelial cells. This is in tandem with astrocyte mobilisation around the site of injury [116], which remains for up to a year post injury [117]. TGF-β is also implicated in immune dysregulation disorders such as Multiple Sclerosis (MS), in which a drastic increase in TGF-β expression was observed during an MS attack and increased TGF-β circulation observed in patients afflicted with the disease [118]. Furthermore, increased TGF-β activity was also observed in neurodegenerative diseases. It has been posited that the increase in TGF-β is a protective response against neuronal loss that occur in these diseases. Evidence compiled in a review of the role of TGF-β in neurodegeneration seems to support this view as aging and chronic inflammation impair TGF-β/Smad signalling and promotes overactivation of microglia, which implicates loss of TGF-β signalling in plaque and tangle formation [119]. Indeed, post-mortem analysis of Alzheimer’s and Parkinson’s disease patients showed that TGF-β ligands were elevated in cerebrospinal fluid [120,121].

The potential for TGF-β to be applied in a therapeutic context has been considered in the context of oncology where anti-TGF-β treatment has been proposed to target tumour cells and microenvironment [122]. This concept is expanded upon in studies where various TGF-β inhibitors have been tested against fibrotic diseases and cancer, showing encouraging results [123]. The possible negative effects of TGF-β inhibition therapy have been examined as well, and it was found that adverse changes to major organs and lymphocyte function were limited in mice treated with anti-TGF-β antibodies chronically [124]. Even a lifetime inhibition of TGF-β receptors has not produced significant adversities in mice [125]. However, a high degree of inhibition has been observed to result in epithelial hyperplasia and carcinoma in certain conditions [126]. Similarly, TGF-β upregulation has also been discussed as a potential treatment to Alzheimer’s Disease [127], but relatively little has been discussed regarding the therapeutic potential of TGF-β outside of the context of cancer.

Although there is a link between neurogenesis and depression, several studies [128,129,130] indicated this was an indirect relationship. In support of neurogenesis playing a role in the aetiology of depression, clinical studies showed a correlation between depression and neuronal degeneration in the hippocampus [131,132] and reduced hippocampal volume [93,94,95,133]. Furthermore, some established depression treatments, such as 5-HT, tianeptine, and electroconvulsive therapy, have been observed to stimulate neurogenesis [70,133,134]. Preclinical studies using animal models showed antidepressants could induce neurogenesis [135,136], which is necessary to mediate the antidepressant effect as the abolition of neurogenesis resulted loss of antidepressant function [137]. Consistent with these observations, exposure to stressors was found to inhibit neurogenesis, which recovered after antidepressant treatment [82]. All these studies provide a convincing case for the involvement of neurogenesis in the aetiology of depression. However, this narrative is complicated by some studies that showed neurogenesis is not correlated with depressive-like symptoms, or is not a causal factor in the development of depression and associated behaviours [138]. Furthermore, antidepressant treatments might act through neurogenesis-independent mechanisms to achieve disease attenuation [139]. Yet other studies showed that neuronal proliferation, which is a standard measure of neurogenesis, may be preserved despite decreased cell survivability [140]. Taken together, our current understanding points to the hypothesis that suppressed neurogenesis predisposes to depression but does not necessarily produce depressive symptoms [141,142,143].

Analysis of human hippocampal neurogenesis showed that neurogenesis takes place at a roughly equivalent rate to that in middle-age rodents [144] and persists even into old age [145], indicating that neurogenesis plays a role in normal brain function in humans. The relationship between hippocampal neurogenesis and mood dysregulation is still unclear, although stress appears to impair hippocampal neurogenesis and is associated with depressive behaviours [146]. This observation is in line with the findings that hippocampal neurogenesis regulated the endocrine stress response, and that loss of neurogenesis in the dentate gyrus resulted in hypersecretion of corticosteroids in response to stress leading to reduced clearance of glucocorticoids [147]. Nonetheless, discrepancies across studies make it difficult to define the precise role of hippocampal neurogenesis in the aetiology of depression. The proposed concept of the ‘neurogenic interactome’ attempts to reconcile these discrepancies. The theory posits three key elements: (1) Interconnectivity between the hippocampus and regions such as the HPA and limbic system, (2) interrelations between mood and memory, and (3) complex interplay among heterogenous components of the neurogenic niche influencing the observed response in behaviour and drug effectiveness [73].

The observation that the inhibition of neurogenesis does not replicate the stress-induced hippocampal volume loss or behavioural changes indicates that neurogenesis is not the sole mechanism in the manifestation of depressive behaviours, and it is likely that stress affects other contributory factors. Regarding the loss of neurogenesis, previous work found it could be induced by autophagic cell death of neural stem cells. It was shown that deletion of Atg7, an important molecule in the formation of autophagosomes, resulted in neuroprotective effects in chronic restraint stress (CRS) and cortisol injection [148]. This may explain why the inhibition of neurogenesis was insufficient to reproduce the morphological changes seen in stressed animals. Clinical research showed that depression-related changes to hippocampal volume largely occurred in the posterior hippocampus [149,150]. In rodents, the dorsal hippocampus also showed stress-related volume losses [151], while the ventral hippocampus was largely unaffected [71]. These changes were suggested to involve variation in glucocorticoid receptor distribution in the dorsal and ventral hippocampus [152], and variation in amygdala input to the dorsal and ventral hippocampus [153,154]. However, the volume changes were not shown to be the result of reduced neurogenesis, indeed, other factors such as loss of interneurones and microvasculature [155,156], and reduction and shrinkage of astrocytes in the hippocampus [157,158,159] were shown to play a part.

In conclusion, the TGF-β pathway is involved in many functions and is required for embryonic viability; however, its role in neurogenic mechanisms and their implication on mood regulation are largely unknown. Based on the available evidence, the TGF-β pathway potentially exerts effects on neurogenesis via the canonical pathway. These effects may in turn play a role in the development of mood disorders such as depression and pathological anxiety. Inactivation of Smad3 in animal models of depression induced by chronic restraint stress reveals TGF-β/Smad3 signalling together with non-canonical pathway components such as TAK1 and Erk play potential roles in hippocampal neurogenesis.

## Figures and Tables

**Table 1:** TGF-β canonical and non-canonical signalling mechanisms in behavioural and physiological changes.

Signalling Molecule	Models	Behavioural and Physiological Changes	Mechanisms	References
TGF-β	Receptor inhibition in vivo (C57BL6 Mouse)	Increased neurogenesis	Reduction of inflammatory response mediated by B2M through attenuation of pSmad3 activity	[50]
	Knockout in vivo (C57BL6 Mouse)	Increased neuronal degeneration and microgliosis	TGF-β-related decrease in laminin-reduced survivability and increased susceptibility to apoptosis	[46]
	Chronic upregulation of TGF-β1 in vivo (C57BL6 Mouse)	Decreased immature hippocampal neurones and neurogenesis	Induced early cell cycle exit of neural progenitor cells	[54]
	Exogenous upregulation of TGF-β2 in vitro (Sprague-Dawley Rat Hippocampus)	Induction of evoked post-synaptic currents and inhibition of miniature post-synaptic currents	TGF-β-related upregulation of CREB in hippocampal neurones	[35]
	Receptor knockout in vivo (C57BL6 Mouse)	Reduction of immature neurones and neurogenesis	Increased expression of pro-apoptotic effectors; decreased expression of anti-apoptotic effectors	[55]
Smad3	Knockout in vivo (C57BL6 Mouse)	Reduction of Neurogenesis	Disruption of neuronal proliferation and migration	[49]
		Inhibition of long-term potentiation	Impairment of NMDA activity by Smad3-related increase in GABAergic signalling	[52])
		Rostral increase of proliferative cells; caudal decrease in neurogenesis	Potential compensatory mechanism in rostral DG to maintain cell numbers; increased apoptosis at intermediate cell stage reduces neurogenesis	[58]
		Decreased neuronal viability following injury	Disruption of Smad3 signalling in astrocytes	[59]
		Accelerated wound closure and decreased activation of microglia	Reduced expression of MCP-1 and reduced leukocyte activity	[60]
	Transient knockdown in vivo (Chick Embryo)	Decreased neurogenesis	Preferential activation of Smad2 targets due to loss of Smad3 activity	[48]
Smad2	Transient knockdown in vivo (Chick Embryo)	Increased neurogenesis	Preferential activation of Smad3 targets due to loss of Smad2 activity	[48]
Braf	Knockout in vivo (129S1/Sv + C57BL6 Mouse)	Increase of depressive-like behaviour in adults, decrease of anxiety in juveniles; reduction of dendritic spine growth	Disturbance of Erk/MAP signalling and alteration of serotonergic transmission	[33]
CREB	Inhibition by dominant negative mutant in vivo (129SvEv + C57BL6 Mouse)	Anti-depressant effects mediated by increase in neurogenesis	Potential mCREB interaction with non-CREB targets	[38]
		Decreased granule cell proliferation	Disruption of cAMP-CREB signalling	[39]
	Conditional knockout in vivo (129SvEv + C57BL6 Mouse)	Impairment of performance in spatial retention	Upregulation of CREB	[36]
	Transient overexpression of CREB in vivo (Sprague-Dawley Rat)	Reduction of depressive-like behaviours	Improved adaptation due to CREB-related regulation of granule cells	[41]
Gadd45b	Knockout in vivo (Mouse)	Reduction of the effectiveness of ECT in inducing neurogenesis and dendritic spine growth	Attenuation of Gadd45b-mediated demethylation in regulatory regions of BDNF and FGF1	[61]
JMJD3	Knockout in vivo (Mouse)	Disruption of neuronal migration and reduction of neurogenesis	Reduction of Dlx2 activation and H3K27me3 demethylation	[62]
			Disruption of TGF-β/Smad signalling cascade	[63]
MAPK	Transient inhibition in vivo (Mouse)	Increased behavioural despair and reduced effectiveness of anti-depressant treatment	Disruption of MEK-ERK signalling	[31]
TAK1	Knockdown in vivo (Mouse)	Reduction of axonal length	Impairment of JNK activity mediated by TAK1	[64]
TrkB	Conditional knockout in vivo (Mouse)	Impairment of neurogenesis; resistance to anti-depressant treatment	Blockade of BDNF signalling	[65]

Abbreviation: B2M, β2 microglobulin; NMDA, N-methyl-D-aspartic acid; GABA, gamma-aminobutyric acid; DG, dentate gyrus; MCP-1, Monocyte chemoattractant protein-1; FGF1, Fibroblast growth factor 1; Dlx2, distal-less homeobox 2.
